# Immunomodulation by IVIg and the Role of Fc-Gamma Receptors: Classic Mechanisms of Action after all?

**DOI:** 10.3389/fimmu.2014.00674

**Published:** 2015-01-21

**Authors:** Sietse Q. Nagelkerke, Taco W. Kuijpers

**Affiliations:** ^1^Department of Blood Cell Research, Sanquin, University of Amsterdam, Amsterdam, Netherlands; ^2^Department of Pediatric Hematology, Immunology and Infectious Disease, Emma Children’s Hospital at the Academic Medical Center, University of Amsterdam, Amsterdam, Netherlands

**Keywords:** IgG, Fc gamma receptors, immunomodulation, IVIg, mechanisms of action

## Abstract

Intravenous IgG (IVIg) contains polyclonal immunoglobulin G (IgG) from thousands of donors. It is administered at a low dose at regular intervals as antibody replacement therapy and at a higher dose as immunomodulatory treatment in various auto-immune or auto-inflammatory diseases. The working mechanism of immunomodulation is not well understood. Many different explanations have been given. During the last decade, we have focused on classical antibody binding via the Fc-domain of the IgG molecules to the common IgG receptors, i.e. the Fcγ receptors (FcγRs). Variation in the genes encoding human FcγRs determines function as well as expression among immune cells. As described here, NK cells and myeloid cells, including macrophages, can express different FcγR variants, depending on the individual’s genotype, copy number variation (CNV), and promoter polymorphisms. B-cells seem to only express the single inhibitory receptor. Although these inhibitory FcγRIIb receptors are also expressed by monocytes, macrophages, and only rarely by NK cells or neutrophils, their presence is unlikely to explain the immunomodulatory capacity of IVIg, nor does the sialylation of IgG. Direct IVIg effects at the level of the activating FcγRs, including the more recently described FcγRIIc, deserve renewed attention to describe IVIg-related immunomodulation.

## Introduction

Intravenous IgG (IVIg) is a blood product containing polyclonal immunoglobulin G (IgG) isolated and pooled from thousands of donors. IVIg is mainly used in two clinical situations. It is administered at a low dose at regular intervals as antibody replacement therapy in primary immunodeficiencies such as agammaglobulinemia and common variable immunodeficiency (CVID), or acquired immunodeficiencies where IgG plasma concentrations have become very low. In this context, suppletion essentially functions to provide the recipient with a repertoire of protective antibodies against a range of predominantly bacterial infections.

On the other hand, IVIg can also be used at a high dose as an immunomodulatory treatment in auto-immune or auto-inflammatory diseases, of which a handful are FDA- and EMEA-approved, including Kawasaki disease and immune thrombocytopenia (ITP). In this respect, IgG administration has also been used off-label for a growing number of additional diseases, including hematologic, dermatologic, and neuromuscular disorders. A list of indications for treatment with IVIg is provided in Table 1. Many but certainly not all of these diseases involve (auto)-antibody responses, questioning the actual working mechanism of IVIg under conditions where auto-antibodies have not yet been shown to be involved.

**Table 1:** **Overview of indications for treatment with IVIg**.

**IVIg as substitution therapy**
Primary immunodeficiency disease
Chronic lymphocytic leukemia
Pediatric HIV infection
Common variable immunodeficiency
**IVIg as immunomodulatory therapy**
Inflammatory disorders
Kawasaki’s disease
Transplantation
Kidney transplantation involving a recipient with a high antibody titer or an ABO-incompatible donor
Allogeneic bone marrow transplantation
Graft-versus-host disease
Hematologic disorders
Immune thrombocytopenia
Auto-immune hemolytic anemia
Auto-immune neutropenia
HIV-associated thrombocytopenia
Neonatal alloimmune thrombocytopenia
Severe anemia associated with parvovirus B19
Dermatologic disorders
Bullous pemphigoid
Epidermolysis bullosa acquisita
Mucous-membrane (cicatricial) pemphigoid
Pemphigus vulgaris
Toxic epidermal necrolysis or Stevens–Johnson syndrome
Neuromuscular disorders
Birdshot retinopathy
Chronic inflammatory demyelinating polyneuropathy
Multifocal motor neuropathy
Guillain–Barré syndrome
Lambert–Eaton myasthenic syndrome
Myasthenia gravis
Opsoclonus–myoclonus
Polyradiculoneuropathy
Refractory dermatomyositis
Refractory polymyositis
Relapsing–remitting multiple sclerosis

Even though IVIg is widely used and has proven to be an effective treatment for many diseases, the exact immunomodulatory mechanism(s) have remained elusive. Several mechanisms by which IVIg may exert its anti-inflammatory effects have been proposed over the past decades (1), listed in Table 2. These mechanisms are not necessarily mutually exclusive, and may act in concert to modulate the immune system. Furthermore, different mechanisms may be at work in the different diseases for which IVIg is administered. In this review, we describe the different theories that may explain the immunomodulatory effect of IVIg, with a special interest in the actions of IVIg in ITP, being the first disease for which the usefulness of IVIg as an immune-modulatory agent was discovered (2).

**Table 2:** **Potential immunomodulatory mechanisms of IVIg**.

**Fc-mediated mechanisms**
1. Blockade of activating FcγR by saturation via high-dosed IVIg making them less available for auto-antibodies in oligo- or polymeric complex with their (auto)antigen
2. Upregulation of the inhibitory FcγRIIb by sialylated IgG Fc
3. Increased clearance of pathogenic antibodies by saturation of the neonatal FcR (FcRn)
4. Tipping the cellular balance from pro- to anti-inflammatory reactivity by modulating dendritic cells (DCs)
5. Reducing responses to IFN
6. Inhibition of the complement cascade by sequestering complement away from the deposited auto-antibodies
**Fab-mediated mechanisms**
7. Neutralization of various agents (similar to mAb), including chemokines, inflammatory cytokines, and apoptosis-inducing molecules, including FasL
8. Neutralization of auto-antibodies by anti-idiotype Abs – often claimed but never proven to effectively explain the anti-inflammatory potential

Many of the theories aiming to explain the working mechanism involve Fc gamma receptors (FcγRs), which are the main receptors for IgG and therefore very likely to be involved in the working mechanisms of IVIg. Therefore, we start with an introduction of human FcγRs, followed by a description of the potential working mechanisms of IVIg, discussing how IVIg can shape immune responses by altering or interfering with FcγR expression and function.

## Fc Gamma Receptors

FcγRs are receptors for (IgG), the most abundant of five classes of Ig. IgG consists of a Fab (fragment, antigen-binding) region, which determines specificity to specific antigens, and a constant region, which is the Fc (fragment, crystallizable) region, which (among other functions) mediates the effector functions of IgG, including the interactions with their major receptors, the FcγRs. These receptors are found on almost all immune cells (Table 3) and, upon binding of IgG, mediate a wide range of cellular responses, such as phagocytosis of IgG-opsonized microorganisms or immune complexes, antibody-dependent cellular cytotoxicity (ADCC), activation of the NADPH oxidase, and the release of cytokines.

**Table 3:** **Expression of FcγRs on different cell types**.

	FcγRI	FcγRIIa	FcγRIIb	FcγRIIc	FcγRIIIa	FcγRIIIb
B-cells	**−**	**−**	**+**	**−**	**−**	**−**
T cells	**−**	**−**	**−**	**−**	**−**	**−**
NK cells	**−**	**−**	Genotype-dependent^a^	Genotype-dependent^b^	**+**	**−**
Dendritic cells	**+**	**+**	**+**	Genotype-dependent?^b^	**−**	**−**
Macrophages	**+**	**+**	+	Genotype-dependent^b^	**+**	**−**
Monocytes	**+**	**+**	Subsets	Genotype-dependent^b^	Subsets	**−**
Neutrophils	Induced	**+**	Genotype-dependent^c^	Genotype-dependent^b^	**−**	**+**
Eosinophils	Induced^d^	**+**	**−**	**−**	**−**	Induced^d^
Platelets	**−**	**+**	**−**	**−**	**−**	**−**

*Expression of FcγRs on different cell types, derived from our own data [Ref. (4–6), Figure 3, data not shown] as well as review of the literature (3)*.

*^a^Expression of FcγRIIb occurs in NK cells in individuals with a deletion of CNR1 (Figure 2) (4)*.

*^b^Expression of FcγRIIc is dependent on SNPs in exon3 and intron7 of the *FCGR2C* gene, which in most individuals is a non-expressed pseudogene (4, 5)*.

*^c^Expression of FcγRIIb on neutrophils strongly correlates with SNPs in the promoter of the *FCGR2B* gene [promoter haplotype 2B.4 (7), Tsang-a-Sjoe et al., submitted)]*.

*^d^Although FcγRI and FcγRIIIb are definitely absent from eosinophils in the resting state, and are often regarded not expressed by these cells (3), some reports have described inducible expression *in vitro* for FcγRI and FcγRIIIb (8) and also *in vivo* for FcγRIII (9)*.

Importantly, as compared to many innate pattern recognition receptors, human FcγRs are quite different from their murine counterparts in the sense that no clear orthologs can be assigned. As a result, human and murine FcγRs that share nomenclature and CD numbers actually have quite different protein structures, expression patterns, and Ig binding affinities (3). Thus, functional studies on mouse FcγRs can only provide very limited information for understanding the contributions of individual FcγRs to human disease.

### Structure, signaling, and expression

**Figure 1:**
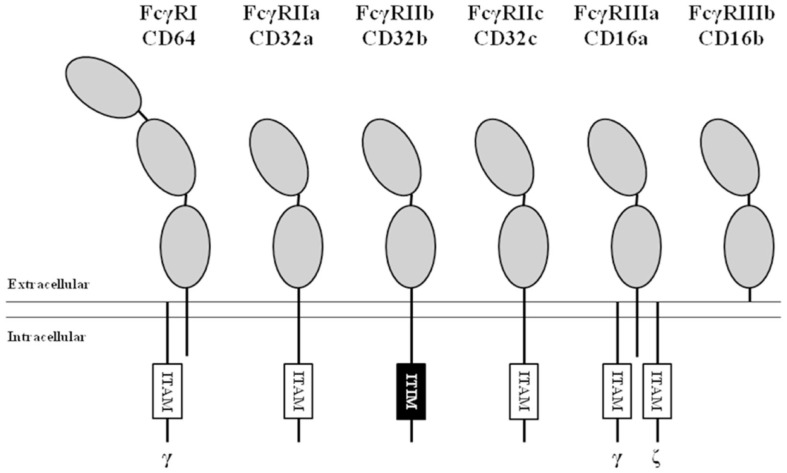
**Nomenclature and structure of human FcγRs**. All human FcγRs are transmembrane proteins, except the GPI-linked FcγRIIIb. The high-affinity FcγRI contains three extracellular (EC) domains (gray ellipses), the low-affinity FcγRs contain two. All isoforms of FcγRII contain either an immunoreceptor tyrosine-based activating (ITAM, white boxes) or inhibitory (ITIM, black box) motif in their α-chain. FcγRI and FcγRIIIa associate with ITAM-containing adaptor proteins such as the Fc receptor common gamma chain (indicated by “γ”) and the CD3 ζ-chain (indicated by “ζ”).

Based on their affinity for monomeric IgG, FcγRs can be divided into the high-affinity FcγRI and the low-affinity FcγRII and FcγRIII (Figure 1). Signaling by FcγR is mediated by immunoreceptor tyrosine-based activating (ITAM) or inhibitory (ITIM) motifs that are present either in the cytoplasmic tail of the receptor itself or in non-covalently associated signaling adaptor proteins, such as the common γ-chain (FcRγ; see below). Aggregation of activating FcγR, i.e., those containing or associated with ITAMs, by binding of multivalent ligands, such as an opsonized pathogen or cancer cell, results in the phosphorylation of ITAM tyrosine residues by Src family protein tyrosine kinases (PTKs), and ultimately leads to activation of cellular responses (10). Aggregation of inhibitory FcγR, i.e., those containing ITIMs, also results in phosphorylation of tyrosine residues by Src family PTKs. In contrast to ITAMs, phosphorylated ITIMs serve as binding sites for phosphotyrosine phosphatases (PTPs), which dephosphorylate other proteins resulting in inhibition of activating pathways (11).

FcγRI (CD64) is a 72 kDa protein that has three extracellular (EC) Ig-like domains, involved in binding of IgG, a transmembrane (TM) domain and a short intracellular (IC) domain of 61 amino acids. The TM domain associates with the FcRγ-chain, an adaptor protein containing an ITAM, to induce signaling and maintain stable expression (12). FcγRI is constitutively expressed by monocytes, macrophages, and dendritic cells and its expression can be induced on neutrophils by stimulation with IFN-γ and/or G-CSF (13, 14). Although there are three genes with various transcripts (15, 16), it is generally believed that only one, the FCGR1A transcript, results in the expression of the classical FcγRIa (CD64).

**Figure 2:**
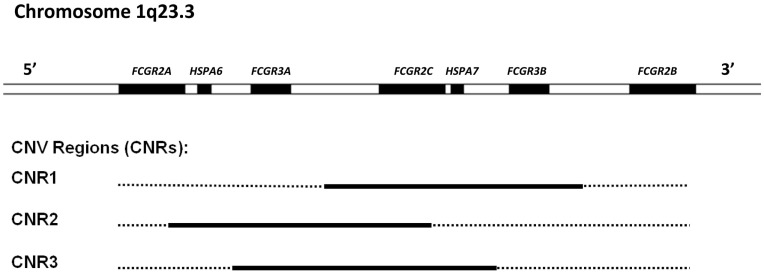
**Overview of the low-affinity FcγR gene cluster and the corresponding CNV**. Three combinations of FcγR genes have been shown to occur in duplication/deletion. Black lines indicate which genes are involved in CNV.

FcγRII (CD32) is actually a collection of three highly homologous proteins, known as FcγRIIa, -b, and -c that all have a molecular mass of ~40 kDa. Their genes are located in one gene cluster at chromosome 1q23.3 (Figure 2). In contrast to FcγRI, the FcγRII proteins have only two IgG binding EC domains. On the other hand, the much larger intracellular domains of FcγRIIa, -b, and -c harbor intrinsic signaling motifs. In contrast to the activating FcγRIIa and -c, FcγRIIb contains an immunoreceptor tyrosine-based *inhibitory* motif (ITIM) (17). As no other FcγR contains or associates with proteins containing ITIMs, FcγRIIb is the only inhibitory FcγR (18).

FcγRIIa is the most widely expressed isoform of FcγRII and is found on monocytes, macrophages, dendritic cells, neutrophils and platelets.

FcγRIIb is highly expressed on B-cells, where it constitutes the only surface-expressed FcγR. FcγRIIb is also expressed, albeit at much lower levels, on a subset of monocytes, on macrophages, and on dendritic cells. Expression of FcγRIIb can also be detected on neutrophils and NK cells, but only in individuals with certain genotypes [Ref. (4), Tsang-a-Sjoe et al., submitted].

**Figure 3:**
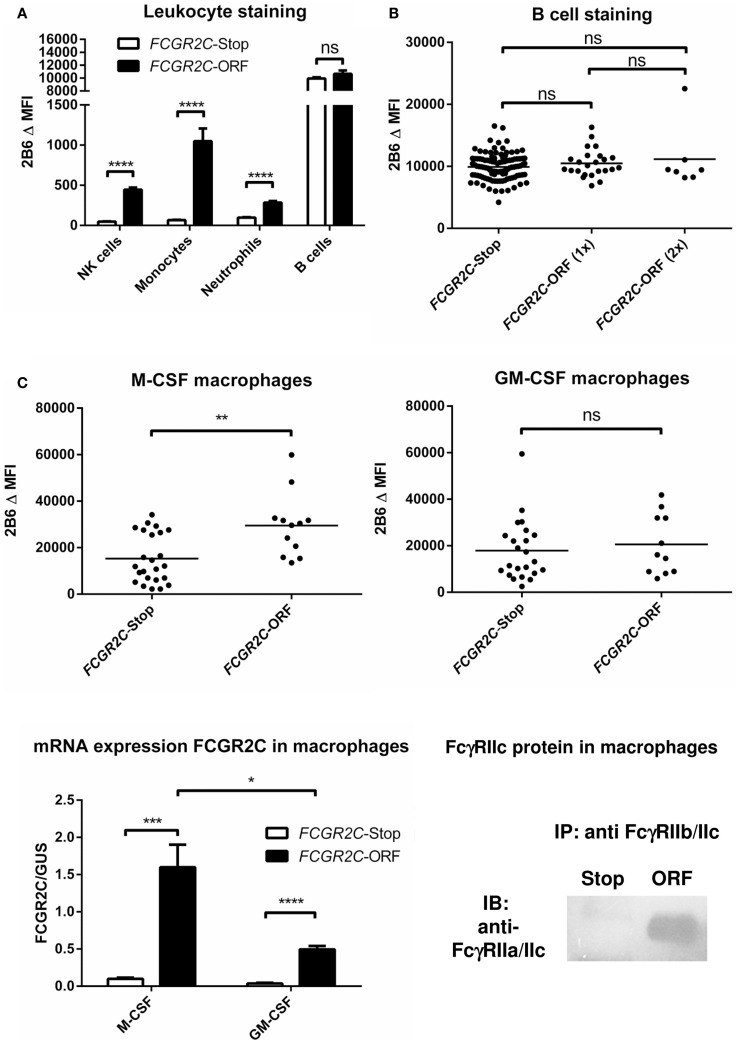
**FcγRIIc expression on various cell types in healthy human subjects**. **(A)** Expression of FcγRIIc and FcγRIIb on circulating leukocytes. Figure adapted from van der Heijden et al. (4), now including measurements from additional individuals. MoAb 2B6 recognizes an extracellular epitope of both FcγRIIb and FcγRIIc, but since *FCGR2C*-Stop individuals cannot express FcγRIIc, the difference in MFI between *FCGR2C*-Stop and *FCGR2C*-ORF individuals can be assumed to derive from expression of FcγRIIc. *FCGR2C*-Stop (individuals with zero copies of *FCGR2C*-ORF) *n* = 105; *FCGR2C*-ORF (including individuals with one and two copies of *FCGR2C*-ORF) *n* = 31. Means + SEM are shown. **(B)** Detailed analysis of MoAb 2B6 staining on circulating B-cells, showing individual measurements, reveals no evidence of expression of FcγRIIc on B-cells. *FCGR2C*-Stop *n* = 105; *FCGR2C*-ORF(1x), individuals with one copy of *FCGR2C*-ORF, *n* = 24; *FCGR2C*-ORF(2x), individuals with two copies of *FCGR2C*-ORF, *n* = 7. **(C)** Expression of FcγRIIc on macrophages. Upper panel: MoAb 2B6 staining on monocyte-derived macrophages cultured for 9 days from 36 healthy individuals, performed as previously described (79). *FCGR2C*-Stop: *n* = 24; *FCGR2C*-ORF, individuals with one (*n* = 11) or two (*n* = 1) copies of the *FCGR2C*-ORF allele. Data are shown for M-CSF (left) and GM-CSF (right) cultured macrophages. Lower left panel: detection of FCGR2C mRNA in monocyte-derived macrophages cultured for 9 days. qPCR with FCGR2C1-specific primers was performed as previously described (5), using cDNA from M-CSF monocyte-derived macrophages as a calibration curve, as described in (79). *FCGR2C*-ORF, individuals with 1 *FCGR2C*-ORF allele, *n* = 3; *FCGR2C*-Stop, individuals with zero copies of *FCGR2C*-ORF, *n* = 4. Means + SEM are shown. Lower right panel: immunoprecipitation of FcγRIIc from M-CSF monocyte-derived macrophages confirms expression of FcγRIIc. Experiment was performed essentially as described in Ref. (4), in this case using MoAb 2B6 to capture FcγRIIc (and FcγRIIb), followed by a specific staining for the intracellular part of FcγRIIc with a rabbit polyclonal antibody against the cytoplasmic tail shared by FcγRIIc and FcγRIIa (25), in macrophages from an individual with zero copies of *FCGR2C*-ORF (Stop), and an individual with one copy of *FCGR2C*-ORF (ORF). Data are representative of three independent experiments with different individuals. For reasons of simplicity, in this figure, individuals with the non-classical *FCGR2C*-ORF allele that is not expressed (4) [*n* = 6 for **(A,B)**, were grouped with *FCGR2C*-stop individuals. Individuals with a deletion of CNR1 (*FCGR2C* and *FCGR3B* genes], which leads to ectopic expression of FcγRIIb on NK cells (4) (*n* = 14), were left out of the analysis of NK cells in **(A)**. Statistical significance was tested by Mann–Whitney *U* test. ns (*p* > 0.05); *p < 0.05; **p < 0.01; ***p < 0.001; ****p < 0.0001. Δ MFI: median fluorescence intensity of MoAb 2B6 minus median fluorescence intensity of isotype control. Some individuals were analyzed more than once at different time points with similar results, means are shown for these individuals.

FcγRIIc has long been considered not to be expressed at all, as its gene (*FCGR2C*) was thought to be a pseudogene (19, 20), and therefore, relatively little was known about the expression pattern of this receptor. In 1998, FcγRIIc was first found on NK cells of individuals with a particular haplotype of the receptor (21), but we now know that – apart from NK cells – it can also be expressed on neutrophils and monocytes in the individuals with the appropriate genotype (4, 5) (Figure 3A). As this activating FcγRIIc is expressed on circulating monocytes of some individuals, it may be expected that expression also occurs on (monocyte-derived) macrophages of these same individuals, but this has not been reported to date. Here, we show for the first time that monocyte-derived macrophages do indeed express FcγRIIc, at least when cultured in the presence of M-CSF (M2 phenotype), as shown by flowcytometry stainings with MoAb 2B6, which recognizes both FcγRIIb and FcγRIIc (Figure 3C). Although these data are difficult to interpret because of the (varying) presence of FcγRIIb on these cells, we can assume the mean difference in MFI between *FCGR2C*-ORF and *FCGR2C*-Stop donors to derive from FcγRIIc. Expression of FcγRIIc specifically was confirmed by qPCR of FCGR2C mRNA and a specific immunoprecipitation using a combination of MoAb 2B6 and a polyclonal antibody that binds FcγRIIc but not FcγRIIb (Figure 3C). Monocyte-derived macrophages differentiated with GM-CSF (M1 phenotype) do not seem to express FcγRIIc based on flowcytometry, although also in these cells, low levels of FCGR2C mRNA could be detected, therefore expression of FcγRIIc cannot be ruled out and may occur in later stages of differentiation of M1 macrophages. Recently, it was proposed that FcγRIIc can also be expressed on B-cells (22). However, evidence of expression of this receptor on B-cells on our own cohort of healthy donors was not confirmed on B-cells, irrespective their naïve or memory phenotype (Figure 3B; data not shown).

FcγRIII (CD16), similarly to FcγRII, actually represents a collection of two genes, expressed within the gene cluster at chromosome 1q23.3, each encoding proteins with two EC Ig-like domains (Figures 1 and 2). Due to differences in glycosylation, their molecular masses are in the range of 50–80 kDa. FcγRIIIa is similar to FcγRI in its TM and IC domains. In monocytes and macrophages, this receptor associates with the FcRγ-chain, while in NK cells it associates with the CD3 ζ-chain to induce signaling (26–28). In contrast to FcγRI, association with these adaptor proteins is not only essential for maintaining stable expression but also for targeting the receptor to the cell membrane (28).

FcγRIIIb is a GPI-anchored protein, expressed only on neutrophils and eosinophils. As it does not have a TM domain, it cannot associate with FcRγ or the ζ-chain. Nonetheless, FcγRIIIb has been suggested to induce signaling, although the exact mechanism(s) is still unclear (29, 30), and current believe is that it functions mainly as a decoy receptor (31).

Recently, it has been proposed that a totally different class of receptors, the C-type lectins, may also bind the Fc-region of IVIg (32), and such receptors were termed “type II Fc receptors.” In humans, DC-SIGN was proposed to bind IgG with a sialic acid sugar moiety at the Asn297 N-linked glycosylation site of IgG. However, this interaction could not be reproduced by another group (33), which found no binding at all of IgG-Fc regions to DC-SIGN, regardless of the sialylation status. Therefore, with evidence for the interaction of IgG-Fc and DC-SIGN being currently marginal and not broadly supported in the literature, in our opinion, it remains to be seen whether DC-SIGN should indeed be classified as a true IgG-Fc receptor.

### Genetic variation in FcγR: polymorphisms

The genes encoding the classical FcγRs are highly polymorphic and functionally relevant genetic variations have been described for all low-affinity FcγRs (Table 4).

**Table 4:** **Functionally relevant genetic variation in FcγR**.

Gene	Type	Variants	Functional relevance
*FCGR2A*	SNP	H131, R131	H131 has a higher binding affinity for IgG1 and IgG2 than R131 (34)
*FCGR2A*	Splice site mutation	c.739 + 871A, c.739 + 871G	c.739 + 871A > G leads to splice variant FcγRIIa^exon6*^, which shows increased cellular activation
*FCGR2B*	SNP	I232, T232	I232 inhibits FcγRI as well as B-cell receptor signaling more strongly than T232 (35)
*FCGR2B*, *FCGR2C*	Promoter haplotype	2B.1, 2B.2, 2B.3, 2B.4	2B.2 is linked with an ORF in *FCGR2C* (5).
			2B.4 results in increased transcription of *FCGR2B* (7)
*FCGR2C*	SNP	Q13, Stop13	Q13 leads to an ORF in exon 3 and expression of FcγRIIc (21), when combined with c.798 + 1G
*FCGR2C*	Splice site mutation	c.798 + 1G, c.798 + 1A	c.798 + 1A leads to mis-splicing and lack of expression of FcγRIIc (4)
*FCGR3A*	SNP	V158, F158	V158 has a higher binding affinity for all human IgG isotypes than F158 (52)
*FCGR3A*	CNV	1, 2, 3, or 4 copies	Copy number relates to expression levels of FcγRIIIa and NK-cell IgG-mediated ADCC (24)
*FCGR3B*	Polymorphic variants	NA1, NA2, SH	NA1 phagocytizes more efficiently than NA2 (40, 57)
*FCGR3B*	CNV	0, 1, 2, 3, or 4 copies	Copy number relates to expression levels of FcγRIIIb and the binding and uptake of IC’s

In *FCGR2A*, encoding for FcγRIIa, a single nucleotide polymorphism (SNP) was first noticed, which results in either a histidine or an arginine at position 131 (H131R) in the IgG binding domain (EC2) (34). FcγRIIa-H131 has a higher binding affinity for IgG1 and especially IgG2, as compared to FcγRIIa-R131, but binding to IgG3 and IgG4 is similar for both variants (38). Functionally, mononuclear cells from FcγRIIa-131HH individuals produce more IL-1beta when stimulated with IgG2 than FcγRIIa-131HR and -131RR individuals (39). Similarly, neutrophils from individuals homozygous for H131 (FcγRIIa-131HH) have been shown to have increased phagocytosis and degranulation in response to serum-opsonized bacteria and increased rosette formation and phagocytosis in presence of IgG3 anti-D sensitized erythrocytes when compared to FcγRIIa-131RR individuals (36, 40).

*FCGR2B* also exists in two allelic variants, encoding for FcγRIIb containing either an isoleucine or a threonine at position 232 in the TM domain (35). As this SNP (I232T) does not affect the IgG-binding EC domains, it has no influence on the binding affinity. However, its localization at the TM domain results in differences in downstream signaling and subsequent inhibition of FcγRI signaling in macrophages and BCR signaling in B-cells. In particular, I232 provides stronger inhibitory signaling than T232, and this is caused by the exclusion from lipid rafts of FcγRIIb-T232 (41, 42). As FcγRIIb is the only inhibitory FcγR, it has a central role in the regulation of immune responses. The loss-of-function FcγRIIb-T232 has been linked to susceptibility and/or severity of several auto-immune diseases, particularly SLE (43–45), and also in rheumatoid arthritis (RA) (46) and ITP (47).

Inter-individual variation in FcγRIIb is also found in expression patterns and levels. Similar to the I232T SNP, the important immune-regulatory role for FcγRIIb is also reflected in the observations of aberrant expression levels of FcγRIIb in SLE, RA, ITP, and chronic inflammatory demyelinating polyneuropathy (7, 48–51). As a result of a deletion in the *FCGR* locus that includes *FCGR2C*, *FCGR3B* and is called CNR1, FcγRIIb can surprisingly also be expressed on the surface of NK cells, where it is capable to inhibit killing of target cells in ADCC (4). Expression of FcγRIIb in other cells is hardly affected by this deletion. Furthermore, two SNPs in the proximal promoter of *FCGR2B* and *FCGR2C*, a guanine or cytosine at position −386 and a thymine or adenine at position −120, form four haplotypes of which one (−386G, −120A; 2B.3) has never been found in any individual thus far. In case of *FCGR2B*, the wildtype promoter (−386G, −120T; 2B.1) has a lower transcriptional activity than one of the other haplotypes (−386C, −120A; 2B.4) [Ref. (51); Tsang-a-Sjoe et al., submitted].

In case of *FCGR2C*, only the wildtype and one other promoter haplotype (−386C, −120T; 2B.2) are found. Moreover, the 2B.2 haplotype is linked to another polymorphism in *FCGR2C* (5). This other polymorphism, a SNP in exon 3, determines whether or not individuals can express FcγRIIc at all. This C > T mutation results in either an open-reading frame (*FCGR2C*-ORF, allele frequency ~10–15% in Caucasians) or a stop codon (*FCGR2C*-Stop) (5). Although expression on NK cells is low, it has been shown to be capable of inducing killing of target cells in a redirected ADCC assay (5). Classically, ORF/Stop genotyping of individuals is done based on this SNP alone. However, we have recently found that some individuals carry splice site mutations in intron 7 that introduce novel stop codons, leading to a loss of FcγRIIc expression (4), and genotyping *FCGR2C* should include these novel mutations to provide an accurate prediction for FcγRIIc expression.

The FcγRIIIa-encoding *FCGR3A* gene contains a SNP that results in either a valine or a phenylalanine at position 158 (V158F), located in the second EC domain (52). FcγRIIIa-V158 has a higher binding affinity for all human IgG classes compared to FcγRIIIa-F158 (38). In ADCC assays, NK cells from FcγRIIIa-V158 donors show increased killing of target cells that are opsonized with sub-saturating levels of Rituximab (53).

FcγRIIIb-encoding *FCGR3B* gene exists in three polymorphic variant proteins, NA1, NA2, and SH, which are also known as HNA-1a, -1b, and -1c, respectively (54, 55). FcγRIIIb-NA1 and -NA2 nucleotide sequences differ at five positions [G > C at nucleotide (nt) 141, C > T at nt 147, A > G at nt 227, G > A at nt 277, and G > A at nt 349], with four predicted amino acid differences (R36S, N65S, D82N, and V106I for NA1 and NA2, respectively). As a consequence, the NA2 variant has two additional N-linked glycosylation sites, compared to NA1. The SH variant is identical to NA2 at the five positions that distinguish NA1 from NA2, but differs from both variants at one additional position (C > A at nt 266), resulting in an A78D amino acid change that predicts a change in the tertiary structure of the protein. Additional complexity is added by the discovery of rare individuals carrying other mutations within this gene or different combinations of these nucleotide polymorphisms (37, 56), indicating that the NA1/NA2/SH typing is incomplete. While the binding affinities for IgG1 and IgG3 appear similar between the three variants (38), neutrophils from FcγRIIIb-NA1NA1 individuals bind and phagocytize IgG-opsonized bacteria and red blood cells more efficiently than those from FcγRIIIb-NA1NA2 and -NA2NA2 individuals (40, 57).

### Gene copy number variation

Besides being polymorphic, some of the low-affinity *FCGR* genes are subject to gene copy number variation (CNV). Although several large-scale studies on CNV have suggested that human *FCGR2A* and *FCGR2B* are candidate genes for CNV (58–61), our group has shown previously that this is not the case. In fact, CNV in the *FCGR* locus is restricted to *FCGR2C*, *FCGR3A*, and *FCGR3B* (24). It occurs in three different combinations: *FCGR3A/FCGR2C* (two possibilities with slightly different borders to the CNV region), and *FCGR2C/FCGR3B* (Figure 2).

Copy number variation translates into differences in expression levels of FcγRIIc (in case of *FCGR2C*-ORF), FcγRIIIa, and FcγRIIIb, with more gene copies leading to a higher receptor expression (and vice versa) (21, 62, 63). In case of FcγRIIIa, the level of expression on NK cells is, at least for 1 versus 2 copies, related to the level of killing of target cells in (redirected) ADCC assays (24). Increased expression of FcγRIIIb leads to higher binding and uptake of immune complexes (ICs) (64).

As is the case with polymorphic variants, CNV in *FCGR* genes is associated with several auto-immune diseases. Our group has previously shown that *FCGR2C-*ORF predisposes for ITP. The SNP in exon 3 causing an open-reading frame instead of a stop allele of *FCGR2C*, results in the expression of FcγRIIc and thus behaves as if it were CNV of *FCGR2C*-ORF. However, individuals can have an increased CNV at this locus of three *FCGR2C*-Stop alleles without increased risk since only the ORF allele was shown to predispose to ITP (5).

Although we could not find an association with various disease cohorts (24), an increased copy number of *FCGR3A* has been observed in anti-glomerular basement membrane antibody disease (anti-GBM disease) (65).

In contrast, a low copy number of *FCGR3B* has been shown to be a risk factor for SLE, even when linkage disequilibrium between *FCGR3B* CNV and FcγR SNPs that have previously been shown to be associated with SLE is taken into account (66–70). Similar associations have been reported for Sjögren’s syndrome (67), systemic sclerosis (71), and RA (72, 73), although other reports have shown no association with RA (67, 74).

## Potential Working Mechanisms for the Immunomodulatory Effect of IVIg

Potential mechanisms can be divided into two categories, being dependent on either the Fc part or the Fab part of the IgG molecule. For some indications, such as ITP, clinical studies with human subjects have in fact already revealed what part of the IgG molecule is effective, as preparations with only Fab fragments of IVIg were not effective (75), whereas purified Fc fragments did have a good clinical effect (76). Thus, we know that at least for ITP, the immunomodulatory effect is Fc-mediated, although this may be different for other indications. Here, we focus most on Fc-mediated modes of action, and will briefly discuss Fab-mediated mechanisms.

## Fc-Mediated Working Mechanisms

### Blockade of activating FcγR by saturation as a result of high IgG concentrations

Administration of IVIg greatly increases the total concentration of IgG in the recipients’ plasma and extracellular fluid, and with such an increase, more FcγRs may be bound by circulating non-complexed IgG, thereby saturating the FcγRs and making them less available for auto-antibodies in oligo- or polymeric complex with their (auto)antigen. The idea that especially the low-affinity FcγRs can be blocked by their monomeric ligand *in vivo* may at first hand seem surprising, but it has been shown in the past that also low-affinity receptors bind monomeric IgG (77), indicating that some “low-affinity” FcγRs are not so low-affinity, and maybe should be better named “medium-affinity,” especially in the case of FcγRIIa and FcγRIIIa (38). Greatly increasing the concentration of monomeric IgG above the normal plasma levels may shift the equilibrium toward a situation in which too many FcγRs are occupied for proper functioning – which may in part explain the immunomodulatory actions of IVIg under some of the conditions for which IVIg is used. Saturation of activating FcγRs was one of the first theories that was formulated to explain the working mechanism of IVIg (2), and this “classic” mechanism has for a long time been assumed as the most plausible explanation for the effect of IVIg in ITP (23, 78). Circumstantial evidence for this theory derives from observations that IgG preparations with increased affinity for FcγRs appear to have an increased effect (6, 79), and that in all diseases in which an immunomodulatory effect is wanted, high doses of IVIg are needed. Nevertheless, there is no formal proof for this concept, and although it has never been disproven, focus has shifted away from this theory as other explaining theories arose.

### Upregulation of the inhibitory FcγRIIb as a result of sialylated IgG-Fc

Over the past decade, the prevailing theory for the working mechanism of IVIg in most immunomodulatory situations has become that IVIg induces an upregulation of the inhibitory FcγRIIb on effector cells. More specifically, a fraction of IVIg, i.e., the IgGs containing a sialic acid sugar residue at the end of the N-linked glycosylation site at Asn297, would be responsible for this effect by binding to SIGNR1 (mouse), or its human ortholog DC-SIGN, inducing various signaling cascades ultimately leading to the upregulation FcγRIIb. This theory has recently been excellently reviewed in Ref. (80). However, the major problem with this theory is that many findings could not be reproduced by other research groups. For instance, we have recently found that FcγRIIb is not upregulated in human macrophages in response to IVIg, but nevertheless, these macrophages respond very well to IVIg treatment, being inhibited in phagocytosis (79). The role of FcγRIIb in ITP treatment by IVIg was also questioned in mouse studies (81). Similarly, we found that IgG-Fc sialylation was not important for the effect of IVIg on human macrophages (79), and many groups have recently published evidence that IgG-Fc sialylation of IVIg is not required for the immunomodulatory effects (82–85). As mentioned before, even the binding of sialylated IgG-Fc to DC-SIGN could not be reproduced (33). Furthermore, essentially all the evidence supporting this theory derives from murine studies, which may not be translated to the human situation, as mice and humans extensively differ in FcγR expression. Many of the murine studies describing this theory for instance use a model for arthritis, but IVIg has never proven to be a useful therapy in treating arthritic patients (86–88).

On the other hand, glycosylation may still be important, influencing the binding affinity IgG molecules to the various FcγRs. For instance, the binding affinity of FcγRIIIa is undoubtedly influenced by the level of fucosylation of the Fc-domain of IgG, a notion that may help to develop new, afucosylated IgG treatment options (89, 90). An important question will be whether the anti-inflammatory properties are directly influenced by afucosylated IgG or IgG otherwise modified in their glycosylation status.

### Increased clearance of pathogenic antibodies by saturation of the neonatal FcR

FcRn is a receptor expressed by human endothelial cells to recycle plasma IgG, extending its half-life in the circulation (91, 92); saturating this “rescue-receptor” with a high dose of IVIg may shorten the half-life of all IgG including harmful auto-antibodies. Interestingly, for a number of diseases in which IVIg therapy is beneficial, plasmapheresis, aiming to remove pathogenic auto-antibodies by replacing the patients’ plasma with donor plasma is also a good option. This is for instance the case in Guillain Barre syndrome (93, 94). On the other hand, plasmapheresis is not effective in ITP (95, 96), and so apparently, rapid removal of auto-antibodies is not effective in ITP, suggesting that the effect of IVIg in ITP must be exerted in a different way.

### Balance from pro- to anti-inflammatory reactivity by modulating dendritic cells

Recent data have confirmed the expression of FcγRII isoforms, including FcγRIIb (97, 98) on dendritic cells (DCs), which may help to explain the subsequent steps in which inhibition of autoantibody release by B-cells, inhibition of T-helper (Th)1 and Th17 differentiation, and enhancement of CD4^+^FoxP3^+^ regulatory T cells (Treg), helps to modulate certain unwanted (auto)inflammatory responses. IVIg may be able to reset the balance at the level of DCs, involving not only the classical IgG receptors but also non-classical lectin-like surface molecules, as has been repeatedly proposed during the last decade (33, 99). We should emphasize that the relevance of such mechanisms and non-classical IgG receptors remain to be shown in humans for the IVIg-associated effects for immunomodulation.

### Reducing responses to IFN

A recent report showed an increased expression of type I interferon response genes in ITP patients, which was rapidly reduced in patients after receiving IVIg, leading to decreased expression of FcγRIII on monocytes, thereby altering the balance between activating and inhibiting FcγRs (100). The relevance of interferons in such responses is unclear, as interferon response genes have been found in various diseases, among which is SLE, and sometimes independent of clear-cut reaction to treatment and clinical response to therapy (101, 102).

#### Inhibition of the complement cascade

Inhibition of the complement cascade by sequestering complement away from the deposited auto-antibodies as suggested in dermatomyositis (103). On the other hand, with the recent insight that IgG is only able to activate complement by means of generating hexamers, and not as single molecules or dimers (104), it is less likely that complement scavenging roles can realistically be involved in the anti-inflammatory IVIg-mediated effects.

## Fab-Mediated Working Mechanisms

### Neutralization of auto-antibodies by anti-idiotype Abs

One of the first explanations for the anti-inflammatory effect of IVIg was that there are anti-idiotypic antibodies present in the IVIg that neutralize the pathogenic auto-antibodies. This theory is often claimed but to our knowledge has only been proven to effectively explain the anti-inflammatory potential of IVIg in the case of neutralizing antibodies to coagulation factor VIII, which could be inhibited by anti-idiotypic antibodies in IVIg (105).

### Neutralization of endogenous chemokines, inflammatory cytokines, and apoptosis-inducing molecules

Apart from the known microbial antigen-specific binding properties, IgG preparations also contain neutralizing and clearance-enhancing antibodies that may switch a proinflammatory trigger into an anti-inflammatory condition. This suggests that healthy individuals from which plasma is collected and pooled for therapeutic IgG preparations already contain autoreactive “natural” antibodies at low levels in their blood. The infusion of such natural antibodies into the patient may be sufficient to reset certain diseases by the cross-reactive capacity of such natural “auto”antibodies (106–110).

## Role of Fc-Gamma Receptors in Shaping the Immune Response in Relation to the Potential Actions of IVIg

**Figure 4:**
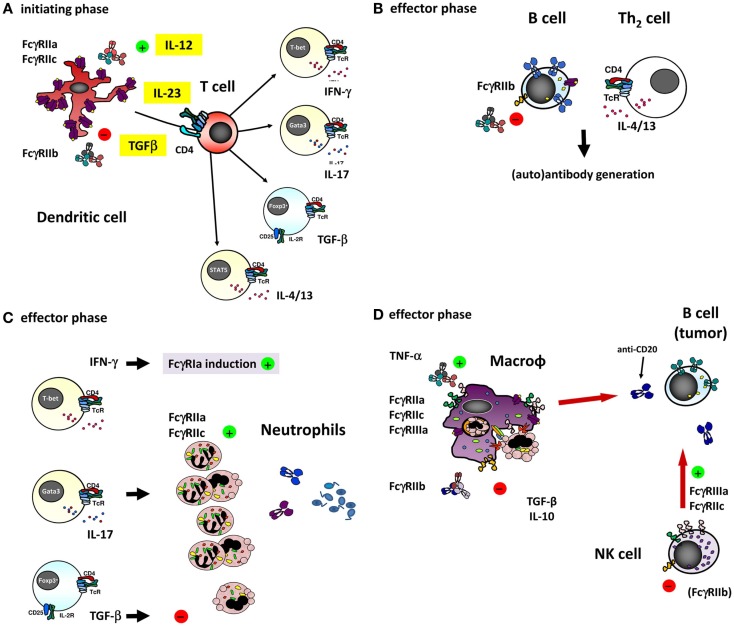
**The different ways of how IVIg may influence an immune response involving (auto)-antibodies by interfering with the function of the different FcγRs on various cell types**. **(A)** dendritic cells (DCs) play a major role in the initiating phase of an antibody response, and expression and function of different FcγRs on these cells may influence this response in several ways. For instance, ligation of FcγRIIa on DCs has been shown to influence the cytokine secretion by DCs in response to various TLR ligands, promoting Th17 responses (98). Furthermore, expression of FcγRIIa on myeloid DCs was recently shown to be downregulated by IVIg in an IL-33 dependent manner, which resulted in a Th2 cytokine response (97). As the co-stimulatory signals derived from DCs determine T cell function, they are ultimately important in shaping an antibody response, as well as the extent of generating a neutrophil-driven response. T cells themselves are crucial in determining the differentiation of most B-cells necessary for the antibody response, but are not known to express any classical FcγRs or other IgG receptors themselves, suggesting that they may be influenced by IVIg in indirect ways only. **(B)** Differentiation of B-cells into antibody producing cells is a major component of an (auto)-antibody response, and involves somatic hypermutation, class switch recombination and plasma cell differentiation. B-cells express high levels of FcγRIIb, and ligation of this receptor has been shown to dampen B-cell receptor signaling. Recently, it was proposed that also FcγRIIc is expressed on B-cells in some individuals, and may influence the immune response in vaccination settings, although it appears that the expression of FcγRIIc on B-cells is very limited if present at all, especially when compared to expression of FcγRIIb (see also Figure 3B). **(C)** Once an (auto)-antibody is formed, innate effector cells such as neutrophils are determinants of tissue damage at the site of deposited immune complexes by release of toxic components such as reactive oxygen species or proteases. Neutrophils express FcγRIIa and FcγRIIc in some individuals, which can contribute to tissue damage and inflammation, and may be influenced by IVIg. Following the afferent immunological phase, the effector phase of activated neutrophils may be in part driven by the presence of certain Th subsets. The “inflammation-promoting” Th17 activity may be counterbalanced by Tregs inducing immunosuppressive properties by TGF-β1 as indicated by expression of neutrophil IL-10 and IL-6, indoleamine 2,3-dioxygenase (IDO), heme oxygenase-1, and “suppressor of cytokine signaling-3” (SOCS3) (113), although the production of these cytokines by human neutrophils remains controversial. **(D)** In case of natural or therapeutic antibody responses against cellular targets, these targets can be eliminated by cells of the innate immune system, for instance through antibody-dependent phagocytosis by macrophages, or antibody-dependent cellular cytotoxicity by NK cells (or monocytes and neutrophils). These responses are mediated by FcγRs on these effector cells, which could be influenced by IVIg in a direct way, i.e., saturating activating FcγRs by binding to them, or in a more indirect way as a result of upregulation of the inhibitory FcγRIIb on effector macrophages (discussed in the text).

Clearly, the different genetic FcγR variants may not only be a risk factor for the development of some auto-immune diseases but may possibly also influence the efficacy of treatment of these diseases by IVIg. Indeed, some SNPs can be overrepresented in Kawasaki disease (KD) patients that respond well to IVIg therapy, but not in the non-responders. Among KD patients, patients who respond well to IVIg have been reported to more often carry the promoter polymorphism 2B.4 in *FCGR2B* and the FcγRIIIb-NA1 when compared to non-responders (111, 112). In both these cases, the balance between activating and inhibitory receptor signaling is altered. A shift toward the inhibitory side of the balance increases the efficacy of IVIg treatment, while a shift toward the activating side shows the opposite effect. Connections of IVIg efficacy in KD and/or other auto-immune diseases with other polymorphisms or CNV in FcγR have not been found to date. Given the growing number of diseases in which IVIg therapy is successfully used and the number of possible working mechanisms that involve FcγR, it does not seem unlikely that more such connections exist. The ways in which IVIg may interact with FcγRs to exert its immunomodulatory actions are multiple, since many different FcγRs are expressed by different immune cells. An overview of the potential interactions is given in Figure 4. One special case is the FcγRIIc, discussed in more detail below.

### FcγRIIc as common denominator in tipping pro- or anti-inflammatory balances?

As mentioned above, a SNP in exon 3 of *FCGR2C* determines whether or not individuals can express FcγRIIc at all (5). Although expression on NK cells is low, it has been shown to be capable of inducing killing of target cells in a redirected ADCC assay (5). We could also detect FcγRIIc expression on neutrophils and monocytes in individuals with an ORF allele (Figure 3A) (4). When investigating surface FcγRIIc expression on monocyte-derived macrophages skewed to either M1 or M2 phenotype, the M-CSF-cultured cells were clearly expressing FcγRIIc (Figure 3C).

Surprisingly, a recent report also found FcγRIIc expression on B-cells (22). Upon transfection into a murine B-cell line, the co-ligation of FcγRIIc with the BCR resulted in enhanced and more sustained tyrosine phosphorylation of the key B-cells signaling components Syk and BLNK. In contrast, the engagement of FcγRIIb with the BCR and its activation caused a reduced level of Syk and BLNK phosphorylation. Antibodies generated upon immunization in this transgene mouse model were found to be enhanced, coinciding with a higher level of B-cell activation (22). In a cohort of about 300 individuals of unknown ethnicity the levels of Ab against a neoantigen (Anthrax protein) were tested (22). At the earliest time points in the vaccine study donors homozygous for the *FCGR2C-*ORF allele [i.e., two alleles (*n* = 11)] showed higher Ab levels at 4 weeks (*p* < 0.02) but not any longer at 8 weeks (22). The more common single-ORF donors were not included.

Although interesting, direct proof of FcγRIIc expression in human B-cells is lacking, as the protein was only specifically detected in EBV-transformed B-cell lines (22). Stainings with an antibody detecting both FcγRIIb and FcγRIIc showed a difference between Stop and ORF donors (22), suggesting some expression of FcγRIIc in primary and memory B-cells. However, we performed similar stainings in a much larger cohort and did not detect such differences. Hence, we must conclude that the expression of FcγRIIc on primary B-cells is at most marginal if present at all.

In fact, the myeloid expression on macrophages and DCs of FcγRIIc may be held responsible for the earlier peak in Ab generation in *FCGR2C*-ORF-positive donors. Not only the macrophage as effector mechanism in immune responses could be relevant for adaptive immunity including Ab generation but also the afferent part of adaptive responses may be involved, as may also be suggested for FcγRIIc-expressing human DCs in *FCGR2C-*ORF individuals. Thus, we hypothesize that FcγRIIc may be a subtle but relevant genetic factor in the fine balance between health and disease, including the way the immune response will shape the adaptive repertoire as indicated by the immunization studies mentioned above (22) (Figure 4, overview).

## Conclusion

The Fc-gamma Receptors constitute the major receptors for human IgG. There may be low-affinity receptors with lectin-like binding properties that have been suggested to bind a fraction of IgG depending on IgG glycosylation, such as sialylation, but definite proof awaits further study. The beneficial effects of sialylated IVIg in mice are model-dependent, and evidence that sialylation of IgG plays a role in humans has not been generated thus far. Evidence for a prominent role of DC-SIGN in mediating the anti-inflammatory activity of IVIg in humans is also lacking. Thus, clinical application of sialic acid-enriched IVIg in humans is supposed not to be superior to conventional IVIg. In fact, the classically proposed mechanism of IVIg saturating the FcγRs still appears to be the most logical explanation for the immunomodulatory effects in at least some diseases for which it is indicated, for instance in ITP. However, different mechanisms may be at work in other inflammatory diseases for which IVIg is used, and it is not unlikely that different mechanisms act in concert. Fab-mediated IVIg actions may be relevant for some indications, but clinical studies have in the past ruled out Fab-mediated mechanisms to be important for ITP. Knowledge on whether the immunomodulatory effect of IVIg for a given indication is Fc- or Fab-mediated may become very important if alternative (i.e., not donor-derived) sources of IgG are to be used in the future. When the effects of IVIg are Fc-mediated, the polyclonal aspect of IVIg is clearly not important, and recombinant IgG preparations may suffice, which can then be specifically modified to enhance function. On the other hand, for indication in which the effects are Fab-mediated, the polyclonality is likely to be very important, and recombinant preparations can only be successful if the relevant clones can be identified and expanded for therapeutic IgG production. However, the results of the ITP studies preclude further clinical trials with Fab-only or Fc-only preparations for other indications, as this may withhold patients a currently effective therapy – which clearly is unethical. Hence, it will remain difficult to determine the relative importance of Fab and Fc for indications other than ITP in the human situation.

Although the sialylation of IgG-Fc appears not to be relevant for its immunomodulatory effect, the glycosylation status of IgG may still be important for its function, as the properties of IVIg preparations can for instance be dependent on the level of fucosylation, having effect on the binding affinity to FcγRs. Further studies will help to resolve the effects of the different glycosylation moieties of IgG-Fc on the interactions with the various receptors for IgG-Fc, and the relevance of these interactions for IVIg function. Finally, FcγRIIc is one novel activating IgG receptor that may add to tip the balance of immune responses, which needs further in-depth study, using proper detection methods to obtain evidence by genotyping and biochemistry.

Concluding, an enormous increase in insight has been generated during the last decade that may help to improve IgG therapy, either as supplement or anti-inflammatory approach. Further studies related to glycosylation may be highly relevant in this respect, but the immunomodulatory effects of IVIg seem not to be determined by the level of sialylation as studied in-depth over the last decade.

## Conflict of Interest Statement

The authors declare that the research was conducted in the absence of any commercial or financial relationships that could be construed as a potential conflict of interest.
